# Acute Lower Respiratory Tract Infections in Soldiers, South Korea, April 2011–March 2012

**DOI:** 10.3201/eid2005.131692

**Published:** 2014-05

**Authors:** Jung Yeon Heo, Ji Eun Lee, Hye Kang Kim, Kang-Won Choe

**Affiliations:** The Armed Forces Capital Hospital, Seongnam, South Korea (J.Y. Heo, J.E. Lee, K.-W. Choe);; Catholic University College of Medicine, Seoul, South Korea (H.K. Kim)

**Keywords:** adenovirus, adenovirus infections, humans, military personnel, soldiers, military, acute respiratory tract infections, lower respiratory tract infections, LRTI, viral respiratory disease, viruses, South Korea, Republic of Korea, etiology, epidemiology, pneumonia, tracheobronchitis, bronchiolitis

## Abstract

During April 2011–March 2012, we retrospectively reviewed medical records for South Korea soldiers to assess the etiology and epidemiology of acute viral lower respiratory tract infections. Adenovirus was the most commonly identified virus (63.2%) and the most common cause of pneumonia (79.3%) and hospitalization (76.6%); 3 soldiers died of adenovirus-related illness.

Respiratory infections are the most common cause for hospitalization of soldiers. In the US military, respiratory infections account for 25%–30% of all hospital admissions for infectious diseases (*1*). Adenovirus is the most common cause of acute respiratory infection in soldiers, particularly among new recruits in basic training camps (*2*,*3*). A study among US military showed that in the early phases of basic training, ≈10% of new recruits were infected with adenovirus, and among recruits with pneumonia, 90% of the cases were caused by adenovirus (*4*). In US military, adenovirus serotypes 4 and 7 have been historically the most common cause of febrile respiratory illness (*5*–*8*). Since 1971, the US military has orally vaccinated new recruits with live adenovirus vaccine; this vaccination has become the primary preventive strategy against respiratory diseases caused by adenovirus (*9*,*10*).

In spring 2006 in South Korea, a high adenovirus prevalence of 61% was found among military recruits with mild respiratory disease (*11*). More recently in South Korea, deaths caused by severe pneumonia were reported among the military, and an outbreak of acute respiratory disease caused by adenovirus occurred in an army training camp (*12*,*13*). We hypothesized that, although there may be some differences in etiologic agents by geographic location, the major cause of acute respiratory disease in South Korean military recruits is most likely adenovirus, as observed in the US military. To describe the viral etiology, clinical features, and epidemiologic characteristics of acute lower respiratory tract infections (LTRIs) among the South Korean military, we retrospectively reviewed the medical records of soldiers who were identified with an acute LTRI.

## The Study

The study was conducted during April 2011–March 2012 at the Armed Forces Capital Hospital, a military referral hospital in Seongnam, South Korea. We retrospectively reviewed all medical records with International Classification of Diseases, Tenth Revision, Clinical Modification codes indicating acute LTRI (pneumonia, tracheobronchitis, or bronchiolitis); 622 patient records met the criteria and were reviewed. General characteristics of the study population and the military hospital, as well as the method used for classifying clinical diagnoses of acute LTRI, are available in the Technical Appendix. 

Nasopharyngeal swab specimens were collected from 207 (33.3%) of the 622 patients with an acute LTRI. Within 3 days, the specimens were tested (GClabs, Yongin-si, South Korea) for 12 respiratory viruses by multiplex reverse transcription PCR; methods are described in the Technical Appendix. Respiratory virus infections were confirmed for 87 (42.0%) patients: adenovirus (55 [63.2%] patients), influenza A virus (26 [29.9%] patients), influenza B virus (4 [4.6%] patients), rhinovirus group A (3 [3.4%] patients), and parainfluenza virus (2 [2.3%] patients). Co-infection with adenovirus and rhinovirus group A was observed in 3 patients. For all confirmed cases of viral infection, we performed an epidemiologic analysis and analyzed the clinical manifestations and prognosis for the hospitalized patients.

The clinical diagnoses for acute viral LTRI cases included 58 cases of pneumonia, 25 cases of tracheobronchitis, and 4 cases of bronchiolitis. Pneumonia was most commonly caused by adenovirus (46 [79.3%] cases) (Table 1). Tracheobronchitis was most commonly caused by influenza A virus and adenovirus (14 [56.0%] and 9 [36.0%] cases, respectively). Two cases each of influenza A virus infection and parainfluenza virus infection were noted among the 4 patients with bronchiolitis. The monthly distribution of the identified viruses is shown in the online Technical Appendix Figure.

**Table 1:** Causes of and diagnoses for acute lower respiratory tract infections in soldiers, South Korea, April 2011–March 2012

Virus identified^✻^	Clinical diagnosis, no. (%) patients			
	Pneumonia, n = 58	Tracheobronchitis, n = 25	Bronchiolitis, n = 4	Total, N = 87
Adenovirus†	46 (79.3)	9 (36.0)	0	55 (63.2)
Influenza A	10 (17.2)	14 (56.0)	2 (50.0)	26 (29.9)
Influenza B	2 (3.5)	2 (8.0)	0	4 (4.6)
Rhinovirus group A†	2 (3.5)	1 (4.0)	0	3 (3.4)
Parainfluenza	0	0	2 (50.0)	2 (2.3)

*A total of 207 nasopharyngeal swab specimens were collected from soldiers with acute lower respiratory infections. Within 3 days of collection, the specimens were tested against 12 respiratory viruses at GClabs (Yongin-si, South Korea) by using Seeplex RV12 ACE Detection (Seegene, Seoul, South Korea). †Three patients were co-infected with rhinovirus group A infection and adenovirus.

Of the 87 patients with an LTRI, 64 were hospitalized. Among these 64 patients, 49 (76.6%) had adenovirus infections, 13 (20.3%) had influenza A or B virus infections, and 2 had bronchiolitis caused by parainfluenza virus infection. Table 2 shows the clinical features of the 62 patients hospitalized for adenovirus or influenza A or B virus infection, among whom the mean age was 19.6 ± 1.1 years (adenovirus patients) and 20.1 ± 2.0 years (influenza patients). Except for a female patient infected with influenza A virus, all patients were male. The proportion of new military recruits was significantly higher among patients with adenovirus infection than among patients with influenza A or B virus infection (p = 0.011). There was no difference in the clinical signs and symptoms and radiographic findings of patients infected with influenza virus and adenovirus (Technical Appendix). Mechanical ventilation was required for 6 patients in whom severe pneumonia developed; all 6 patients had adenovirus infection and 3 died (Technical Appendix).

**Table 2:** Demographic, clinical, and laboratory findings for soldiers hospitalized with acute lower respiratory infections, South Korea, April 2011–March 2012

Variable	Soldiers hospitalized for infection with		p value*
	Adenovirus, n = 49 (79.0%)	Influenza A or B virus, n = 13 (21.0%)	
Demographic characteristics			
Age, mean y ± SD	19.63 ± 1.16	20.15 ± 2.03	0.232
Male sex	49 (100.0)	12 (92.3)	0.210
Military rank, no. (%)			0.011
New recruit	32 (65.3)	3 (23.1)	
Active-duty soldier	17 (34.7)†	10 (76.9)	
Clinical characteristics, no. (%)			
Fever >5 d	27 (55.1)	3 (23.1)	0.061
Cough	47 (95.9)	11 (84.6)	0.191
Rhinorrhea	29 (59.2)	7 (53.8)	0.729
Sputum	32 (65.3)	7 (53.8)	0.447
Sore throat	30 (61.2)	8 (61.5)	0.984
Dyspnea	9 (18.4)	2 (15.4)	1.000
Nausea/vomiting	8 (16.3)	3 (23.1)	0.685
Diarrhea	13 (26.5)	2 (15.4)	0.493
Chest pain	5 (10.2)	1 (7.7)	1.000
Laboratory findings ± SD			
Leukocyte count (cell/μL)	6,529 ± 2,643	8,110 ± 2,331	0.054
Hemoglobin (g/dL)	14.0 ± 0.9	13.5 ± 1.1	0.384
Platelet count (10^3^cell/μL)	156 ± 29	201 ± 26	<0.001
C-reactive protein(mg/dL)	12.0 ± 3.0	8.5 ± 2.3	<0.001
Radiograph findings, no. (%)			
Consolidation	20 (40.8)	2 (15.4)	0.112
Peribronchial infiltration	26 (53.1)	8 (61.5)	0.585
Effusion	9 (18.4)	1 (7.7)	0.673
Normal	3 (6.1)	3 (23.1)	0.100
Length of hospital stay, mean d ± SD	17.1 ± 4.2	14.3 ± 4.1	0.036
Required mechanical ventilation, no. (%)	6 (12.2)	0	0.328
Died, no. (%)	3 (6.1)	0	1.000

*p<0.05 was considered significant. The statistical analyses used in this study are described in the online Technical Appendix (wwwnc.cdc.gov/EID/article/20/5/13-1692-Techapp1.pdf). †Among the 17 hospitalized active-duty soldiers with adenovirus infection, those ranked as privates were the most common (11/17 [64.7%]). All privates who were found to have adenovirus infection had been relocated to advanced training sites after graduating from the 6-week basic military training course, which suggests that adenovirus might have spread to secondary training sites through recruit redeployment.

## Conclusions

Among the viruses identified as causing acute LTRI in South Korean soldiers, adenovirus was the most common, causing 63.2% of the cases. In addition, adenovirus was identified in 79.3% of pneumonia cases. These findings are similar to those in studies among US military (*1*–*4*). 

Adenovirus infection was more common among new recruits in the South Korean military. However, the infection was also confirmed in all 11 (64.7%) privates among the 17 active duty soldiers at advanced training sites (Table 2). In the South Korea military, adenovirus might have spread to secondary training sites through recruit redeployment, similar to the experience in the US military (*14*).

Cases of severe pneumonia requiring mechanical ventilation were not observed among patients with influenza virus infection; however, 3 of the 6 patients with severe adenovirus-associated pneumonia died. This finding indicates that adenovirus infection is a major cause of death among soldiers with severe respiratory diseases. In 2009, in response to the influenza A(H1N1)pdm09 pandemic, multiplex reverse transcription PCR for respiratory viruses became available in South Korea military hospitals. Before 2009, apart from research purposes, clinical testing for respiratory viruses was not routinely performed at military hospitals. For this reason, South Korea military physicians were unaware that adenovirus was a major cause of respiratory disease among soldiers. Because of the lack of an effective treatment for adenovirus infection, vaccination against adenovirus has been introduced for persons, such as military recruits, at high risk for infection.

Our study has certain limitations. First, we were unable to confirm the serotype of adenovirus for positive case-patients. Because military physicians were unaware that adenovirus was a major cause of acute respiratory disease, additional serotyping was not performed at the time of diagnosis. Second, the period of study was only 1 year. The level of an epidemic varies at different times, thus requiring longer study periods to observe patterns. Third, we retrospectively reviewed medical records of patients with acute LTRI in a central referral military hospital; thus, the study patients may not be entirely representative of South Korea soldiers with acute LTRI. Last, there was a sampling bias with regard to the molecular assay; the test may have been performed only in cases of severe illness or when recommended by clinicians. Nevertheless, our results suggest that among South Korea soldiers with acute LTRIs, those with adenovirus rather than other respiratory infections had more severe clinical outcomes.

Further studies are required to determine the serotype(s) of adenovirus causing infection among the military, and epidemiologic surveillance for adenovirus is needed. In addition, studies on the effectiveness of adenovirus vaccine should be considered.

Technical Appendix Methods and results for a study of acute lower respiratory tract infections in soldiers, South Korea, April 2011–March 2012.This originally said (11/49 [22.4%]) but was changed to reflect the number of privates among the active-duty soldiers rather than the number among the total number of soldiers hospitalized with adenovirus infection.
