# Supplementary material for: Acute Lower Respiratory Tract Infections in Soldiers, South Korea, April 2011–March 2012
Source: Emerg Infect Dis. 2014 May;20(5):875–7. doi: 10.3201/eid2005.131692 (PMC4012816; doi:10.3201/eid2005.131692)
Supplement: Technical Appendix — Methods and results for a study of acute lower respiratory tract infections in soldiers, South Korea, April 2011–March 2012.This originally said (11/49 [22.4%]) but was changed to reflect the number of privates among the active-duty soldiers rather than the number among the total number of soldiers hospitalized with adenovirus infection. [file 13-1692-Techapp-s1.pdf]

Article DOI: http://dx.doi.org/10.3201/eid2005.131692

Acute Lower Respiratory Tract Infections in Soldiers, South Korea, April 2011–March 2012

Technical Appendix

Methods

The General Characteristics of the Study Population and the Korean Military Healthcare System

The study population was limited to military recruits and active duty military soldiers, excluding military retirees and supportive personnel. In the Republic of Korea, military service is mandatory for all males aged 18 years and above. Commonly, people join the military around the age of 20 years. They are enlisted for 21 months, including 6 weeks of basic training camp, followed by 3 months of private, 7 months of private first class, 7 months of corporal, and 4 months of lance corporal service. New recruits, i.e., military trainees who undergo basic military training throughout South Korea, are considered to be military personnel for first 6 weeks after they began serving in the military. They are then promoted to private and are relocated for specialty training to advanced training sites for 4 weeks to 2 months before base assignment.

The Korean military healthcare system comprises infirmaries in army divisions and regiments (primary health center); military hospitals in 13 regions across the country (secondary medical facilities); and a single central referral hospital (tertiary medical facility)—The Armed Forces Capital Hospital, a regional central referral hospital.

Classification of Clinical Diagnoses

The clinical diagnoses of acute lower respiratory tract infection (LRTI) were classified according to the description of Denny et al. (*1*). Pneumonia was diagnosed if the patients had rales on physical auscultation or evidence of pulmonary consolidation on radiographs. Tracheobronchitis was diagnosed if patients had cough and rhonchi. Bronchiolitis was diagnosed in patients who had expiratory wheezing with or without tachypnea.

Respiratory Virus Multiplex Reverse Transcription-Polymerase Chain Reaction

For patients who were diagnosed with acute LRTI, nasopharyngeal swab specimens were collected within 24 hours of the hospital visit by using a flocked swab. The collected specimen was stored at 4°C in viral transport media until testing. Specimens were tested in GClabs (a professional laboratory diagnostic company) within 3 days. Total viral nucleic acid was extracted from the specimen using the Chemagic Viral DNA//RNA Extraction Kit (Chemagen Inc., Baesweiler, Germany). cDNA was synthesized using the CapFishing Full-length cDNA Premix Kit (Seegene Inc., Seoul, Korea). Reverse transcription-polymerase chain reaction (RT-PCR) was performed on the synthesized cDNA of influenza viruses A and B, respiratory syncytial viruses A and B, adenovirus, parainfluenza virus types 1 to 3, rhinovirus group A, human coronavirus 229E/NL63, human coronavirus OC 43, and human metapneumovirus by using the Seeplex RV12 ACE Detection kit (Seegene Inc., Seoul, Korea).Respiratory virus multiplex RT-PCR was carried out according to manufacturer's instructions (*2*,*3*). Amplified PCR products were subjected to electrophoresis in 2% agarose gel with ethidium bromide. A positive confirmation was recorded when the band size matched the marker band size of the control.

Statistical Methods

All statistical analyses were performed using the SPSS statistical package (version 18.0; SPSS Inc., Chicago, IL, USA). We used Pearson's chi-square test or Fisher exact test to compare categorical variables, and Student t-test to compare continuous variables.

Results

Characteristics of the Study Patients

A retrospective review of the medical records of 622 study patients showed that the clinical diagnoses were pneumonia in 135 patients (21.7%), tracheobronchitis in 475 patients (76.4%), and bronchiolitis in 12 patients (1.9%). Clinicians ordered respiratory virus multiplex RT-PCR when the cause of acute LRTI was suspected to be a respiratory virus. Respiratory virus multiplex RT-PCR tests were conducted on 87 patients (42.0%) with pneumonia, 113 patients (54.6%) with tracheobronchitis, and 7 patients (3.4%) with bronchiolitis. The military ranks of patients who had been tested by respiratory virus multiplex RT-PCR were as follows: 79 recruits, 54 privates, 25 private first class, 26 corporals, 15 lance corporals, and 8 officers.

Relationship between Military Rank and Clinical Features in Hospitalized Acute LRTI Patients

Private soldier was the most common military rank (11 cases, 22.4%) among the hospitalized active duty soldiers with adenovirus infection, after excluding new recruits (32 cases, 65.3%). All private soldiers who were found to have adenovirus infection were relocated at advanced training sites after graduating from the 6-week basic military training course. However, the military ranks of hospitalized patients with influenza A and B virus infection were as follows: 3 cases (23.1%) of new recruits, 2 cases (15.4%) of private class, and 8 cases (61.5%) of other active duty soldiers.

Clinical Manifestations in Hospitalized Acute LRTI Patients

There was no difference in the clinical symptoms of patients infected with the 2 viruses. Both influenza virus and adenovirus infections were accompanied by upper respiratory symptoms such as rhinorrhea and sore throat in more than half of the patients, and one-fourth of the patients experienced gastrointestinal symptoms such as diarrhea. Regarding laboratory findings, adenovirus-infected patients had lower platelet counts and higher C-reactive protein levels than those infected with the influenza virus. However, there was no difference in the leukocyte counts. With regard to radiographic findings, peribronchial infiltration was the most common abnormal finding in both adenovirus-and influenza-infected patients (53.1% and 61.5%, respectively). Adenovirus-infected patients showed a higher rate of consolidation (40.8%) and pleural effusion (18.4%) than did influenza virus–infected patients, but the difference was not significant.

Prognosis and Cause of Death

The duration of hospital stay was longer for adenovirus-infected patients (mean hospital days, 17.1±.4.2) than for those infected with the influenza virus (mean hospital days, 14.3 ± 4.1) ($p = 0.036$).

There was 1 case of severe pneumonia due to adenovirus infection complicated by myocarditis, and 2 cases of complications from acute respiratory distress syndrome.

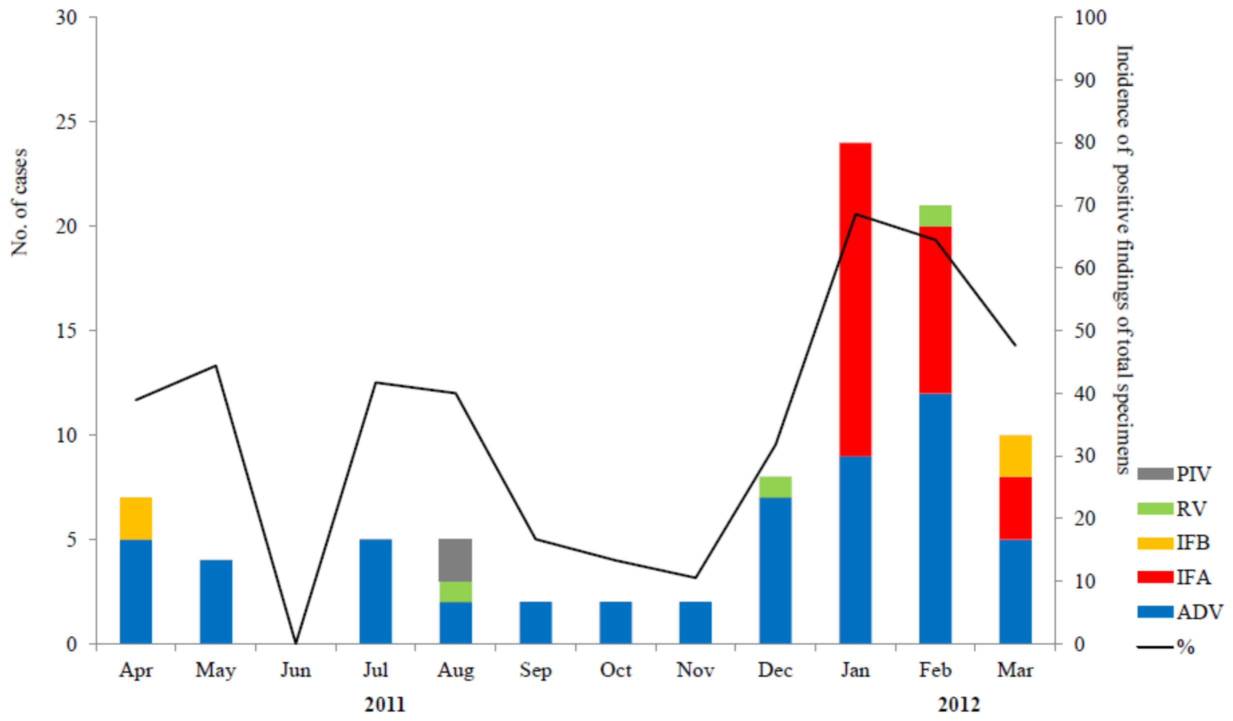

Technical Appendix Figure. Monthly distributions of the identified respiratory viruses and incidence of positive findings among the total specimens in Korean soldiers with acute lower respiratory tract infections. Adenovirus infection was noted throughout the year, but the prevalence was higher between December and May. PIV, parainfluenza virus; RV, rhinovirus group A; IFB, influenza B virus; IFA, influenza A virus; ADV, adenovirus.

References

1. Denny FW, Clyde WA Jr. Acute lower respiratory tract infections in nonhospitalized children. J Pediatr. 1986;108:635–46. PubMed http://dx.doi.org/10.1016/S0022-3476(86)81034-4

2. Yoo SJ, Kuak EY, Shin BM. Detection of 12 respiratory viruses with two-set multiplex reverse transcriptase-PCR assay using a dual priming oligonucleotide system. Korean J Lab Med. 2007;27:420–7. PubMed http://dx.doi.org/10.3343/kjlm.2007.27.6.420

3. Kim SR, Ki CS, Lee NY. Rapid detection and identification of 12 respiratory viruses using a dual priming oligonucleotide system-based multiplex PCR assay. J Virol Methods. 2009;156:111–6. PubMed http://dx.doi.org/10.1016/j.jviromet.2008.11.007
